# Subcortical T1-Rho MRI Abnormalities in Juvenile-Onset Huntington’s Disease

**DOI:** 10.3390/brainsci10080533

**Published:** 2020-08-08

**Authors:** Alexander V. Tereshchenko, Jordan L. Schultz, Ansley J. Kunnath, Joel E. Bruss, Eric A. Epping, Vincent A. Magnotta, Peg C. Nopoulos

**Affiliations:** 1 Department of Psychiatry, Carver College of Medicine at the University of Iowa, Iowa City, IA 52242, USA; alexander-tereshchenko@uiowa.edu (A.V.T.); joel-bruss@uiowa.edu (J.E.B.); eric-epping@uiowa.edu (E.A.E.); Vincent-magnotta@uiowa.edu (V.A.M.); peggy-nopoulos@uiowa.edu (P.C.N.); 2 Department of Neurology, Carver College of Medicine at the University of Iowa, Iowa City, IA 52242, USA; 3 Department of Pharmacy Practice and Science, College of Pharmacy, University of Iowa, Iowa City, IA 52242, USA; 4 Department of Cell Biology and Neuroscience, Rutgers University, New Brunswick, NJ 08854, USA; Ansley.j.kunnath@Vanderbilty.edu; 5 Department of Radiology, Carver College of Medicine at the University of Iowa, Iowa City, IA 52242, USA; 6 Department of Pediatrics, Stead Family Children’s Hospital at the University of Iowa, Iowa City, IA 52242, USA

**Keywords:** juvenile-onset Huntington’s disease, T1-Rho, neuroimaging

## Abstract

Huntington’s disease (HD) is a fatal neurodegenerative disease caused by the expansion of cytosine-adenine-guanine (CAG) repeats in the *huntingtin* gene. An increased CAG repeat length is associated with an earlier disease onset. About 5% of HD cases occur under the age of 21 years, which are classified as juvenile-onset Huntington’s disease (JOHD). Our study aims to measure subcortical metabolic abnormalities in JOHD participants. T1-Rho (T_1ρ_) MRI was used to compare brain regions of 13 JOHD participants and 39 controls. Region-of-interest analyses were used to assess differences in quantitative T_1ρ_ relaxation times. We found that the mean relaxation times in the caudate (*p* < 0.001), putamen (*p* < 0.001), globus pallidus (*p* < 0.001), and thalamus (*p* < 0.001) were increased in JOHD participants compared to controls. Furthermore, increased T_1ρ_ relaxation times in these areas were significantly associated with lower volumes amongst participants in the JOHD group. These findings suggest metabolic abnormalities in brain regions previously shown to degenerate in JOHD. We also analyzed the relationships between mean regional T_1ρ_ relaxation times and Universal Huntington’s Disease Rating Scale (UHDRS) scores. UHDRS was used to evaluate participants’ motor function, cognitive function, behavior, and functional capacity. Mean T_1ρ_ relaxation times in the caudate (*p* = 0.003), putamen (*p* = 0.005), globus pallidus (*p* = 0.009), and thalamus (*p* = 0.015) were directly proportional to the UHDRS score. This suggests that the T_1ρ_ relaxation time may also predict HD-related motor deficits. Our findings suggest that subcortical metabolic abnormalities drive the unique hypokinetic symptoms in JOHD.

## 1. Introduction

Huntington’s disease (HD) is caused by the expansion of cytosine-adenine-guanine (CAG) repeats in the *huntingtin* gene, and the CAG repeat length is associated with an earlier disease onset [1]. About 5% of HD cases occur before the age of 21 years, which are classified as juvenile-onset Huntington’s disease (JOHD) [1]. Adult-onset HD (AOHD) is characterized by involuntary movements (chorea), whereas JOHD often causes bradykinesia [1].

Patients with JOHD experience similar patterns of neurodegeneration as patients with AOHD with some distinct exceptions. Specifically, patients with JOHD have proportionally larger cerebellar volumes relative to controls, and they do not experience thinning of the motor cortex [2] as is seen in AOHD [3]. Since these brain regions are relatively spared, it has been hypothesized that they may play a compensatory role in JOHD. Additionally, both the cerebellum and motor strip play key roles in maintaining motor control, and patients with JOHD experience markedly different motor symptoms compared to patients with AOHD. However, these hypotheses are based solely on volumetric data, and little is known regarding the functional activity of the neurons in these brain regions. T_1_ relaxation in the rotating frame (T_1ρ_) is a novel neuroimaging modality that allows for analysis of biochemical changes in the brain that are undetectable by existing MRI techniques. The use of a spin-locking radio-frequency field increases sensitivity to proton exchange, which is influenced by pH, glucose, glutamate, water, and proteins. T_1ρ_ MRI has previously been used to characterize progressive changes in other neurodegenerative diseases. Specifically, cross-sectional studies have shown that patients with Alzheimer’s disease have increased T_1ρ_ relaxation times in the hippocampus compared to controls [4,5,6,7]. Similar findings of longer T_1ρ_ relaxation times have been reported in patients with Parkinson’s disease [4,8,9], bipolar disorder [10], and multiple sclerosis [11]. These studies all employed cross-sectional designs. T_1ρ_ relaxation times were also shown to increase in the striatum of participants with premanifest patients with AOHD [12].

To the best of our knowledge, T_1ρ_ relaxation times have never been evaluated in patients with JOHD, but may be an important biomarker for progression in this unique subset of patients with HD. Longitudinal changes in the volume of the caudate and putamen have been studied extensively in AOHD and may serve as a valid biomarker for disease progression in that patient population. However, striatal degeneration likely begins very early in life in JOHD and may not change significantly enough over time to serve as a valid biomarker of disease progression for future clinical trials. Here, we aimed to determine if T_1ρ_ relaxation times in subcortical regions of the brain in patients with JOHD were significantly different from healthy controls.

## 2. Materials and Methods

### 2.1. Participants

Participants included in these analyses were enrolled in the Kids-HD and Kids-JOHD studies [2,13,14,15]. These were longitudinal neuroimaging studies that ran in parallel to one another. The Kids-HD study recruited participants between the ages of 6–26 who were at risk for inheriting the gene that causes HD based on their family history (i.e., a parent or grandparent with confirmed HD). The Kids-HD study also recruited healthy control participants. All participants in the Kids-HD study underwent genetic testing for research purposes only. For the present analyses, the control group consisted of participants from the Kids-HD study who had molecular confirmation of a CAG repeat length <36, ensuring that our control group did not include pre-symptomatic patients with AOHD. The Kids-JOHD study recruited participants who had been deemed to have Juvenile-Onset HD (JOHD) by their neurologist and had molecular confirmation of having a CAG repeat length of 36 or above. For these analyses, a participant was considered to have JOHD if they had a total motor score from the Unified Huntington’s Disease Rating Scale (UHDRS) [16] of ≥20 prior to the age of 21. The UHDRS is sensitive to developmental motor changes such that younger children will show higher scores than older children. Therefore, even in a large cohort of children at risk, but who did not inherit the gene expansion, the UHDRS can be as high as 15 [15]. Therefore, a cutoff of 20 on the UHDRS was used to ensure that all participants had confirmed motor-manifest JOHD.

Given the longitudinal nature of these studies, some participants had more than one neuroimaging study conducted. Specifically, there were 11 participants with JOHD that made up 13 visits. Our control group in these analyses consisted of 38 participants that had the necessary neuroimaging done, which included both anatomic (T_1_- and T_2_-weighted) and metabolic (T_1ρ_) images. This consisted of participants who were at risk for inheriting the gene mutation that causes HD but who were found to not carry this gene, as well as healthy control participants without a family history of HD. There were 39 neuroimaging studies amongst this group of 38 participants.

Clinical measures were collected on all participants. As noted previously, all participants received a total motor score as measured using the UHDRS [16]. Motor symptoms were also quantified in the JOHD group using the modified Juvenile HD Motor Rating Scale (JOHDRS) [17]. This rating scale provides additional evaluation of the unique hypokinetic symptoms associated with JOHD. We also calculated a disease burden score (Age × CAG—35.5)) [18] and disease duration (age at time of assessment—age at time of JOHD clinical diagnosis) for JOHD participants.

Signed informed consent was obtained before beginning the study, per the Institutional Review Board at the University of Iowa. All experiments were performed following the guidelines outlined in the Belmont Report. Genetic testing was done for research purposes only. The results were made available to one research team member, and all other team members were blind to these genetic results. The genetic testing results were not revealed to participants or their families.

### 2.2. Data Acquisition

High-resolution magnetic resonance images were collected on a 3T Siemens TIM Trio scanner (Siemens Medical Solutions, Erlangen, Germany) using a 12-channel receiver head coil. Whole-brain T_1_- and T_2_-weighted anatomical acquisitions with a 1.0 mm isotropic spatial resolution were acquired first. T_1_-weighted images were collected using a 3D magnetization-prepared rapid gradient echo sequence with the following parameters: Coronal orientation; field-of-view = 25.6 × 25.6 × 25.6 cm; sampling matrix = 256 × 256 × 256; repetition time (TR)TR/echo time (TE)/inversion time (TI) = 2530/2.8/909 ms; flip angle = 10°; bandwidth = 180 Hz/pixel; and Acceleration Factor (R) = 2 GeneRalized Autocalibrating Partial Parallel Acquisition (GRAPPA). T_2_-weighted images were collected using a 3D variable flip angle spin-echo sequence with the following parameters: TE = 430 ms; TR = 4800 ms; number of echos = 137; bandwidth = 592 Hz/pixel; matrix = 256 × 256 × 170; field-of-view (FOV) = 25.6 × 25.6 × 22 cm; and R = 2 GRAPPA. Next, quantitative parametric imaging was conducted to acquire T_1ρ_ relaxation times. T_1ρ_ mapping was performed using a coronal segmented three-dimensional (3D) gradient echo sequence with spin-lock pulses (TE = 2.5 ms; TR = 5.6 ms, FOV = 220 × 220 × 200 mm; sampling matrix = 128 × 128 × 40; fractional anisotropy (FA) = 10 degrees; integrated parallel acquisition techniques (IPAT) = 2; spin-lock frequency = 330 Hz; spin-lock times = 10 and 55 ms).

### 2.3. Image Analysis

The BRAINS AutoWorkup was used to perform the anatomical image analysis by combining information available from the T_1_- and T_2_-weighted images as described in Pierson et al. [19]. Briefly, the BRAINS AutoWorkup includes the following steps: (1) AC-PC alignment; (2) bias field correction; (3) tissue classification; and (4) anatomical labeling. The anatomical regions-of-interest used for this study include the caudate, putamen, globus pallidus, thalamus, hippocampus, and anterior cerebellum. These regions-of-interest are defined automatically using a neural network-based segmentation [20]. These regions have been shown to have a degree of reliability with a manual rater (Jaccard index ~0.80).

To estimate the T_1ρ_ map, the individual spin-lock images were co-registered using a rigid registration with the Advanced Normalization Tools (ANTs) software [21] to account for subject motion between the two acquisitions. The resulting aligned spin-lock images were then used to calculate a T_1ρ_ map by fitting the 10 and 55 ms spin-lock time (TSL) image signals (S_0_ and S_TSL_) to the following mono-exponential decay model:

(1) $$S_{\mathrm{TSL}}=S_{0}\;\left(e^{-\mathrm{TSL}/T_{1\rho }}\right)$$

The decay model was fit using the “MR Parameter Map Suite” implemented using Insight Segmentation and Registration Toolkit (ITK) [22] and available from the InsightJournal [23]. The resulting rigid body transform was then used to resample the T_1ρ_ map to a 1 mm isotropic resolution using linear interpolation. The T_1ρ_ maps were then thresholded at 400 ms to remove the contribution of cerebrospinal fluid. The defined regions-of-interest generated from the BRAINS AutoWorkup were then used to estimate the mean T_1ρ_ relaxation times for each region from non-zero voxels in the thresholded T_1ρ_ relaxation time maps.

### 2.4. Statistical Analysis

The primary outcomes were mean differences in T_1ρ_ relaxation times from the defined regions-of-interest between groups. We used linear mixed effects models to investigate estimated mean differences in T_1ρ_ relaxation times of the six brain regions above between groups. Our models were controlled for age and sex and included a random effect per participant.

For the secondary analyses, we identified any of the regions-of-interest that demonstrated significant group differences in T_1ρ_ relaxation times from the primary analysis. Amongst those regions-of-interest, we used linear mixed effects regression analyses to investigate the relationship between CAG repeat length and regional brain volumes and T_1ρ_ relaxation times. These models were controlled for age and sex and included a random effect per participant. The models investigating the relationship between brain volume and T_1ρ_ relaxation times were also controlled for intracranial volume (ICV). All of these analyses were performed amongst the JOHD participants only. We then performed a similar analysis to assess the relationship between T_1ρ_ relaxation times and disease burden scores while controlling for sex and a participant random effect. Age was not included as age is used to calculate the disease burden score [18]. Next, we assessed whether T_1ρ_ relaxation times predicted the total motor score, as assessed by the UHDRS and the JOHDR [17], while controlling for age and CAG repeat length. Lastly, we investigated the relationship between the calculated disease duration and T_1ρ_ relaxation times. Again, we performed linear mixed effects regression analyses that controlled for age and sex and a random effect per participant. RStudio (Version 3.6.2, RStudio, PBC, Boston, MA, USA) was used for all statistical analyses and a *p*-value of <0.05 was considered significant for all analyses.

## 3. Results

### 3.1. Primary Outcomes

Participant demographics are tabulated in Table 1.

For the primary analysis, the JOHD group had significantly longer T_1ρ_ relaxation times in the caudate, putamen, globus pallidus, and thalamus compared to the control group (Table 2), indicating significant neuronal damage in these areas in patients with JOHD. However, there were no significant group differences regarding T_1ρ_ relaxation times in the hippocampus and cerebellum, indicating no difference in neuronal damage in these areas.

### 3.2. Secondary Outcomes

**Figure 1:**
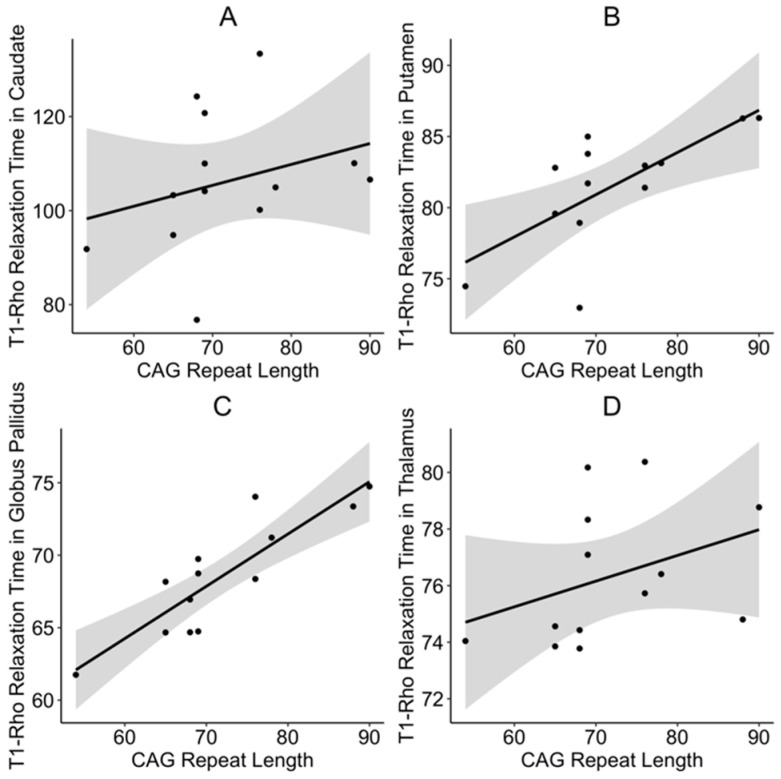
CAG repeat length significantly predicts T_1ρ_ relaxation times in the (**A**) caudate, (**B**) putamen, (**C**) globus pallidus, and (**D**) thalamus. Results show raw data points, the fitted regression line of the model, and 95% confidence interval. CAG: Cytosine-adenine-guanine.

Higher T_1ρ_ relaxation times were associated with lower volumes in the caudate (t = −2.46, *p* = 0.039), putamen (t = −5.63, *p* = 0.0006), and globus pallidus (t = −2.32, *p* = 0.0491). There was a negative relationship between T_1ρ_ relaxation times and volume of the thalamus, but the results did not reach statistical significance (t = −1.92, *p* = 0.0912). Next, we demonstrated significant positive relationships between CAG repeat length and T_1ρ_ relaxation times in the caudate (t = 3.02, *p* = 0.018), putamen (t = 3.73, *p* = 0.006), globus pallidus (t = 7.88, *p* < 0.0001), and thalamus (t = 2.68, *p* = 0.026) (Figure 1A–D). The positive relationship indicates that the higher CAG repeat length is associated with increased neuronal damage in these brain regions.

While the relationship between CAG repeat length and T_1ρ_ relaxation times in the caudate and thalamus were statistically significant, there seemed to be some outlying data that could have influenced the results. Given the small sample size of patients, we performed unplanned follow-up analyses to account for the potential influence of outliers on the results. Specifically, we repeated the analyses using robust linear mixed effects regression using the “robust” package in R. The relationship between CAG repeat length and T_1ρ_ relaxation times in the caudate (t = 2.91, *p* = 0.021) and thalamus (t = 3.18, *p* = 0.012) remained significant after accounting for potential outliers.

**Figure 2:**
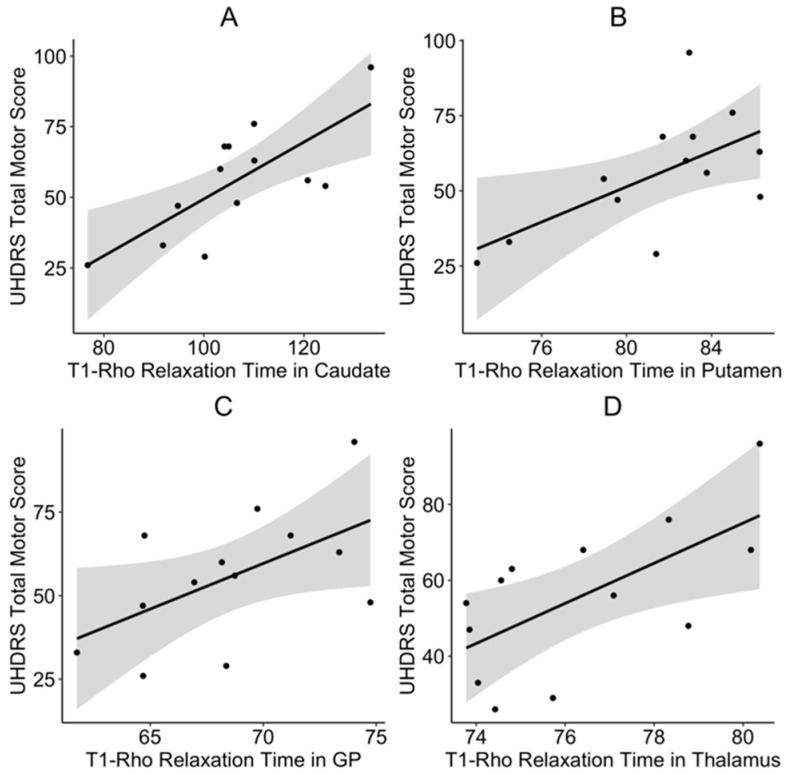
T_1ρ_ relaxation times in the (**A**) caudate, (**B**) putamen, (**C**) globus pallidus, and (**D**) thalamus. Significantly predicted total motor scores as measured by the UHDRS. Results show raw data points, the fitted regression line of the model, and 95% confidence interval. GP: Globus pallidus; UHDRS: Unified Huntington’s Disease Rating Scale.

Next, we assessed the relationship between T_1ρ_ relaxation times and disease burden scores. The disease burden scores significantly predicted T_1ρ_ relaxation times in the caudate (t = 3.97, *p* = 0.003) and thalamus (t = 3.07, *p* = 0.012), but not in the globus pallidus (t = 1.97, *p* = 0.081) or putamen (t = 1.95, *p* = 0.08). We also investigated the relationship between regional T_1ρ_ relaxation times and motor function. We found that higher mean T_1ρ_ relaxation times in the caudate (t = 3.55, *p =* 0.006), putamen (t = 3.26, *p =* 0.011), globus pallidus (t = 3.4, *p* = 0.008), and thalamus (t = 3.29, *p* = 0.042) were positively related to increased UHDRS scores (Figure 2A–D).

Mean T_1ρ_ relaxation times in the caudate (t = 3.08, *p =* 0.013) and putamen (t = 3.51, *p* = 0.007) were also directly proportional to the JOHDRS score, but not in the globus pallidus (t = 1.79, *p* = 0.131) or thalamus (t = 1.09, *p* = 0.357).

Lastly, we identified significant positive relationships between disease duration and T_1ρ_ relaxation times in the caudate (t = 3.58, *p* = 0.008), putamen (t = 3.83, *p* = 0.01), and globus pallidus (t = 3.49, *p* = 0.007), but not in the thalamus (t = 1.09, *p* = 0.304). This suggests that T_1ρ_ relaxation times may be able to track the disease course over time.

## 4. Discussion

In this study, we have utilized a novel neuroimaging method to demonstrate that metabolic abnormalities likely affect the caudate, putamen, globus pallidus, and thalamus in patients with JOHD. However, there were no significant group differences for the mean T_1ρ_ relaxation time in the anterior cerebellum, an area which also controls motor function. Similarly, there were no significant differences in T_1ρ_ relaxation times in the hippocampus between the JOHD and control groups. These results are interesting because the cerebellum is thought to be spared in HD and has even been found to be proportionally enlarged in JOHD [2]. The cerebellum has been hypothesized to play a compensatory role in HD and potentially in JOHD and these results may provide additional support for this theory [24].

The increases in subcortical T_1ρ_ relaxation times were directly related to CAG repeat length in the JOHD group, increasing the likelihood that these findings are related to pathological changes. The disease burden score significantly predicted T_1ρ_ relaxation times in the caudate and thalamus, but did not reach the level of significance for predicting relaxation times in the putamen and globus pallidus, despite a trend in that direction. Again, these findings indicate that T_1ρ_ relaxation times seem to be indicative of disease severity and may be used as a unique measure of disease progression in JOHD. This is further supported by the finding that the longer duration of disease of JOHD was associated with significantly higher T_1ρ_ relaxation times in the caudate, putamen, and globus pallidus.

Patients with JOHD often experience unique motor symptoms. Specifically, patients with JOHD may have less chorea, but more hypokinetic symptoms, including bradykinesia and dystonia [25]. These symptoms can be very difficult to treat and the underlying pathology of this difference between JOHD and AOHD is poorly understood. All of the subcortical regions that we analyzed were significantly and positively associated with the total motor score on the UHDRS. Additionally, relaxation times in the caudate and putamen significantly predicted total scores on the JOHD-specific motor assessment scale. This suggests that metabolic abnormalities in the striatum may drive the unique hypokinetic motor deficits of JOHD.

There are some potential limitations to this study. T_1ρ_ MRI abnormalities in JOHD are not indicative of a specific metabolic dysfunction since it is sensitive to changes in several factors including pH, glucose, glutamate, water, and proteins. Further research using magnetic resonance spectroscopy may investigate specific molecular imbalances in the brain regions identified within this study. Additionally, T_1ρ_ MRI images were captured last in a series of different neuroimaging tests, so JOHD participants with more severe symptoms were not able to complete T_1ρ_ imaging. However, participants with very severe symptoms may have significantly diminished the striatum that is difficult to measure. Finally, the current study was limited by its small sample size due to the rarity of JOHD. As a result, further studies are required to confirm these results in an expanded patient population.

## 5. Conclusions

In conclusion, T_1ρ_ MRI may be a valuable biomarker for monitoring disease progression and evaluating future clinical trials in JOHD. This novel imaging technique allows for high-resolution, quantitative analysis of subcortical metabolic changes. T_1ρ_ MRI abnormalities in JOHD participants were found within the caudate, putamen, globus pallidus, and thalamus, and mean T_1ρ_ relaxation times within these regions were predictive of disease severity and motor deficits.

## Figures and Tables

**Table 1:** Demographics by groups.

	JOHD Group	Controls	*p*-Value
N (Visits)	11 (13)	38 (39)	NA
Male, % (n)	36.4 (4)	39.5 (15)	1
Age (yrs), Mean ± SD	15.39 ± 5.1	15.56 ± 3.82	0.905
CAG Repeats, Mean ± SD	72.82 ± 10.31	19.5 ± 3.85	<0.001
Disease Burden Score, Mean ± SD	540.99 ± 148.11	NA	NA
Disease Duration (yrs), Mean ± SD	3.1 ± 2.61	NA	NA
UHDRS, Mean ± SD	55.27 ± 21.54	NA	NA
JOHDRS, Mean ± SD	14.91 ± 6.71	NA	NA

**Table 2:** Regional T_1ρ_ relaxation times—juvenile-onset Huntington’s disease (JOHD) participants.

Region	Control T_1ρ_ (ms)	JOHD T_1ρ_ (ms)	Beta-Coefficient	*p*-Value
Caudate, mean ± SD	78.34 ± 6.35	106.42 ± 14.21	27.9	<0.001
Putamen, mean ± SD	71.25 ± 2.56	81.79 ± 3.53	10.37	<0.001
Globus pallidus, mean ± SD	64.13 ± 2.79	68.52 ± 4.05	4.62	<0.001
Thalamus, mean ± SD	73.86 ± 2.46	76.38 ± 2.37	2.53	<0.001
Hippocampus, mean ± SD	86.12 ± 8.5	87.65 ± 3.49	1.76	0.484
Anterior Cerebellum mean ± SD	92.12 ± 18.81	94.13 ± 11.55	1.94	0.732
